# Molecular and clinical analyses of 16q24.1 duplications involving *FOXF1* identify an evolutionarily unstable large minisatellite

**DOI:** 10.1186/s12881-014-0128-z

**Published:** 2014-12-04

**Authors:** Avinash V Dharmadhikari, Tomasz Gambin, Przemyslaw Szafranski, Wenjian Cao, Frank J Probst, Weihong Jin, Ping Fang, Krzysztof Gogolewski, Anna Gambin, Jaya K George-Abraham, Sailaja Golla, Francoise Boidein, Benedicte Duban-Bedu, Bruno Delobel, Joris Andrieux, Kerstin Becker, Elke Holinski-Feder, Sau Wai Cheung, Pawel Stankiewicz

**Affiliations:** Interdepartmental Program in Translational Biology & Molecular Medicine, Baylor College of Medicine, Houston, TX USA; Department of Molecular and Human Genetics, Baylor College of Medicine, One Baylor Plaza, Houston, TX 77030 USA; Institute of Informatics, University of Warsaw, Warsaw, Poland; Mossakowski Medical Research Center, Polish Academy of Sciences, Warsaw, Poland; Specially for Children, Dell’s Children’s Medical Center, Austin, TX USA; Departments of Pediatrics and Neurology, University of Texas Southwestern Medical Center, Dallas, TX USA; Neuropediatrics Service, Saint Vincent de Paul Catholic Hospitals Association of Lille, Free Faculty of Medicine, Lille, France; Cytogenetics Service, Saint Vincent de Paul Catholic Hospitals Association of Lille, Free Faculty of Medicine, Lille, France; Laboratory of Medical Genetics, University Hospital, Lille, France; Medical Genetics Center, Munich, Germany

**Keywords:** *cis*-regulation, Satellite repeats, Microhomology-mediated break-induced replication, Microduplication

## Abstract

**Background:**

Point mutations or genomic deletions of *FOXF1* result in a lethal developmental lung disease Alveolar Capillary Dysplasia with Misalignment of Pulmonary Veins. However, the clinical consequences of the constitutively increased dosage of *FOXF1* are unknown.

**Methods:**

Copy-number variations and their parental origin were identified using a combination of array CGH, long-range PCR, DNA sequencing, and microsatellite analyses. Minisatellite sequences across different species were compared using a gready clustering algorithm and genome-wide analysis of the distribution of minisatellite sequences was performed using R statistical software.

**Results:**

We report four unrelated families with 16q24.1 duplications encompassing entire *FOXF1*. In a 4-year-old boy with speech delay and a café-au-lait macule, we identified an ~15 kb 16q24.1 duplication inherited from the reportedly healthy father, in addition to a *de novo* ~1.09 Mb mosaic 17q11.2 *NF1* deletion. In a 13-year-old patient with autism and mood disorder, we found an ~0.3 Mb duplication harboring *FOXF1* and an ~0.5 Mb 16q23.3 duplication, both inherited from the father with bipolar disorder. In a 47-year old patient with pyloric stenosis, mesenterium commune, and aplasia of the appendix, we identified an ~0.4 Mb duplication in 16q24.1 encompassing 16 genes including *FOXF1*. The patient transmitted the duplication to her daughter, who presented with similar symptoms. In a fourth patient with speech and motor delay, and borderline intellectual disability, we identified an ~1.7 Mb *FOXF1* duplication adjacent to a large minisatellite. This duplication has a complex structure and arose *de novo* on the maternal chromosome, likely as a result of a DNA replication error initiated by the adjacent large tandem repeat. Using bioinformatic and array CGH analyses of the minisatellite, we found a large variation of its size in several different species and individuals, demonstrating both its evolutionarily instability and population polymorphism.

**Conclusions:**

Our data indicate that constitutional duplication of *FOXF1* in humans is not associated with any pediatric lung abnormalities. We propose that patients with gut malrotation, pyloric or duodenal stenosis, and gall bladder agenesis should be tested for *FOXF1* alterations. We suggest that instability of minisatellites greater than 1 kb can lead to structural variation due to DNA replication errors.

**Electronic supplementary material:**

The online version of this article (doi:10.1186/s12881-014-0128-z) contains supplementary material, which is available to authorized users.

## Background

Heterozygous point mutations and genomic deletions involving the dosage-sensitive *FOXF1* gene on chromosome 16q24.1 have been reported as causative in patients with a rare, neonatally-lethal developmental lung disorder Alveolar Capillary Dysplasia with Misalignment of Pulmonary Veins (ACDMPV; OMIM 265380) [1-5]. The majority of patients with ACDMPV also have extra-pulmonary anomalies of the gastrointestinal, cardiovascular, or genitourinary systems. In a number of ACDMPV patients negative for mutation and deletion in *FOXF1*, we identified overlapping genomic deletions mapping upstream of *FOXF1*. These deletions enabled us to define an ~60 kb noncoding, evolutionarily-conserved, and differentially-methylated *cis*-regulatory enhancer region that maps ~272 kb upstream of *FOXF1* and harbors lung-specific long non-coding RNA (lncRNA) genes [6,7]. Recently, we demonstrated that the *FOXF1* locus in humans is incompletely paternally-imprinted in the lungs, and that the imprinting likely involves these lncRNAs [6,8]. Moreover, the antisense lncRNA gene, *FENDRR*, located 5 kb upstream of *FOXF1*, was found to associate with the polycomb repressive complex (PRC)2 and negatively regulate *FOXF1* expression [9].

*Foxf1*^-/-^ mice die by embryonic day (e) 8.5 due to vascular abnormalities that stem from defects in mesodermal differentiation and cell adhesion [10]. Approximately 50-90% of the heterozygous *Foxf1*^+/-^ mice, depending on their genetic background, die neonatally due to respiratory failure [11,12]. Endothelium specific homozygous knockout of *Foxf1* using *Tie2*-cre and *Pdgfb-CreER* leads to embryonic lethality around e13.5-e16.5 due to vascular abnormalities in the lung, placenta and yolk sac [13]. Endothelial specific deletion of *Foxf1* causes decreased expression of endothelial genes critical for vascular development, including VEGF receptors *Flt1* and *Flk1*, *Pdgfb*, *Pecam1*, *CD34*, *integrin β3*, *ephrin B2, Tie2* and *Fendrr*. Interestingly, homozygous loss of *Fendrr* in mice was shown to be either embryonic lethal due to heart and body wall defects [14], or perinatal lethal due to multiple defects in lung, heart, or gastrointestinal tract [15].

Previous studies have shown *FOXF1* to be epigenetically inactivated in breast cancer, suggesting its potential role as a tumor suppressor gene [16]. Common variants mapping on chromosome 16q24.1 close to *FOXF1* have also been associated with susceptibility to Barrett’s esophagus in genome-wide association studies [17,18]. Up-regulation of *FOXF1* has been reported in breast cancer [19], rhabdomyosarcoma [20], and in colorectal adenocarcinomas [21]. However, the clinical consequences of constitutively increased dosage of *FOXF1* remain unknown.

Variable Number Tandem Repeats (VNTRs) with repeat units less than nine nucleotides (nt) are referred to as microsatellites, those with repeat units between 10 and 100 nt are defined as minisatellites and those with repeat units greater than 100 nt are termed macrosatellites or megasatellites. VNTRs are extremely unstable, with mutation rates 10-100,000 times higher than non-repeat sequences. They tend to be highly polymorphic, expanding or contracting due to DNA strand replication or recombination slippage [22,23].

Using chromosomal microarray analysis, we identified and molecularly characterized overlapping 16q24.1 duplications harboring entire *FOXF1* in four unrelated families. In addition, we describe an evolutionarily unstable large minisatellite on chromosome 16q24.1, likely responsible for the formation of one of these duplications.

## Methods

### Subject recruitment

Patients 1 and 2 were referred for clinical chromosomal microarray testing in the Medical Genetics Laboratories (MGL) at Baylor College of Medicine (BCM). Patient 3 was referred for genetic counseling to the Medical Genetics Center, Munich, Germany. Patient 4 was reported in Decipher (265898) and was referred for clinical chromosomal microarray testing at the Saint Vincent de Paul Catholic Hospitals Association of Lille, Free Faculty of Medicine, Lille, France. The twelve 16q24.1 non-duplicated control samples were obtained from clinical diagnostic testing at MGL or ACDMPV research study at BCM.

### Patients

Patient 1 is a 4 2/12 year old boy, the third child of a non-consanguineous 20-year-old mother and 22-year old father. Maternal complications prior to delivery included placenta previa. He was born at term via spontaneous vaginal delivery with birth weight of 3.2 kg. He sat unassisted at 5 months and began walking at 9 months. At the age of 28 months, the proband’s height was 93 cm (72^nd^ percentile), weight was 14.5 kg (78^th^ percentile), and head circumference was 29.5 cm (44^th^ percentile). He has mildly dysmorphic facial features, with a broad forehead, sparse eyebrows, mildly low-set ears, and nasal features with small alae, broad tip and broad bridge. He has brachydactyly of fingers and toes. There is one café-au-lait macule on his left flank. He has speech delay, is very limited in the number of words used, and was found to be anxious, hyperactive, uncooperative, aggressive, and impulsive. He tends to sweat a lot, especially at night, and has bedwetting. Every two to three weeks he complains of abdominal pain and feels nauseous, but does not vomit. Pulmonary findings are normal on physical examination, with the lungs clear to auscultation bilaterally without wheezes, rhonchi, or rales. He has had numerous viral upper respiratory infections and was hospitalized around 18 months of age for pneumonia. Both the father and mother are reportedly in good health and have had no learning, speech, or lung problems. Paternal grandparental samples were not available.

Patient 2 is a 13-year-old boy, who based on Diagnostic and Statistical Manual–Fifth edition (DSM-V) was diagnosed with autism, unspecified mood and anxiety disorder, pervasive developmental disorder not otherwise specified and emotional disorder. At the age of 12 years, his height was 141.5 cm (12th percentile), weight was 32.95 kg (10^th^ percentile), and head circumference was 52.5 cm (24^th^ percentile). He is extremely aggressive and has poor social skills. He makes poor eye contact and has fine motor skill delays. There was no evidence of any lung abnormalities except for a remote history of asthma. His father was diagnosed with bipolar disorder.

Patient 3 is a 47-year-old female patient, who at the age of 2 years presented with recurrent vomiting and was subsequently diagnosed with pyloric stenosis, mesenterium commune, and aplasia of the appendix. Her height was 168 cm (49^th^ percentile), weight was 58 kg (47^th^ percentile), and head circumference was 56 cm (71^st^ percentile). Psychomotor development was normal. At the age of 34 years, she had a postpartum iliofemoral deep vein thrombosis on the left side with pulmonary embolism. Factor V Leiden mutation, Prothrombin G20210A mutation, antithrombin deficiency, protein C deficiency and protein S deficiency were excluded. At the age of 42 years, the patient was diagnosed with multiple sclerosis. Her 13-year-old daughter presented with pyloric stenosis with bowel malrotation, aplasia of the caecum and appendix, mesenterium commune, gastroesophageal reflux disease, as well as persistent foramen ovale, unilateral inguinal hernia, and immature hip development. Her height was 162 cm (53^rd^ percentile), weight was 49 kg (42^nd^ percentile), and head circumference was 55 cm (73^rd^ percentile). Dumping syndrome, relative short bowel syndrome, failure to thrive, and dystrophy were noted. Psychomotor development was normal. There was no evidence for any lung abnormalities in the two patients. Patient 3’s father presented with hematemesis and anemia in infancy. Laparotomy at the age of 6 years revealed pyloric stenosis, bowel malrotation, mesenterium commune, and aplasia of the caecum and appendix. In childhood, a caput medusa was noted, and at the age of 18 years, venous ulcers of the legs were observed. Before the age of 50, the father had several iliofemoral deep venous thromboses and inferior vena cava atresia was suspected. At the age of 34 years, a Billroth’s operation II was performed because of a hemorrhagic duodenal ulcer. He was diagnosed with iron-deficiency anemia, protein S deficiency, activated protein C resistance, and renal insufficiency. The father’s DNA was not available for testing.

Patient 4 is a 10 1/12-year-old boy born to healthy and non-consanguineous 23 and 25 year-old parents. His height, weight, and head circumference were 135.5 cm (between the 25^th^ and 50^th^percentile), 29.3 kg (between the 25^th^ and 50^th^ percentile), and 52 cm (between the 10^th^ and 25^th^ percentile), respectively. During the pregnancy, he was diagnosed with a club foot, which was surgically corrected. He presented with speech delay, motor delay, and borderline intellectual disability (ID). No respiratory or cardiac defects were observed. Pedigrees of families 2 and 3 are shown in Additional file 1: Figure S1.

### DNA isolation

Genomic DNA was extracted from peripheral blood via the Puregene DNA isolation kit (Gentra System, Minneapolis, MN, USA) (patients 1 and 2), FlexiGene DNA Kit (Qiagen, Valencia, CA) (patient 3) and QIAamp DNA mini kit (Qiagen, Valencia, CA) (patient 4).

### Array CGH analysis

Chromosomal microarray analysis (CMA) in patients 1 and 2 were performed using array CGH (aCGH) with custom-designed exon targeted aCGH oligonucleotide microarrays V8.1.1 OLIGO, 180K and V9.1.1 OLIGO, 400K, respectively, designed by the MGL at BCM (http://www.bcm.edu/geneticlabs/) and manufactured by Agilent Technology (Santa Clara, CA, USA) as described [24]. In patient 3, aCGH analysis was performed using an Oligonucleotide array (Cytochip v1.0 180K, BlueGnome, Cambridge, GRCh37, Ensembl Release 70) and in patient 3’s daughter, aCGH analysis was done using Illumina SNP-Array (Infinium® CytoSNP-850K; GRCh37). aCGH in patient 4 was performed using a 8 × 60K microarray (Agilent Technology). Fine duplication mapping was performed in patient 1 using aCGH with a custom-designed 16q24.1-specific 720K microarray covering 2-Mb regions flanking *FOXF1* [Roche-NimbleGen (Madison, WI, USA)] as described [25], and in patients 2 and 4 using a custom-designed 16q24.1 region-specific 4 × 180K microarray (Agilent Technology), as described [6].

### FISH analysis

Confirmatory fluorescence *in situ* hybridization (FISH) analyses were performed using the standard procedures.

### Long-range PCR and DNA sequence analysis

The junction fragments of the duplications were amplified using long-range PCR with Takara *LA Taq* Polymerase (TaKaRa Bio USA, Madison, WI, USA) according to the manufacturer’s instructions. Primers were designed using Primer3 software (http://frodo.wi.mit.edu/primer3/), at the apparent head-to-tail duplication boundaries inferred from the 16q24.1 custom-designed array CGH analysis. PCR products were visualized on a 1% agarose gel, purified with ExoSAP-IT (USB, Cleveland, OH, USA) to remove unconsumed dNTPs and primers, and directly sequenced via Sanger method (Lone Star Labs, Houston, TX, USA).

### Microsatellite analysis

Microsatellite markers *D16S539, D16S488, D16S486, D16S520,* and *D16S3074* for parental studies were PCR amplified and analyzed by capillary electrophoresis (SeqWright, Houston, TX), followed by analysis using the GeneMapper software (Applied Biosystems, Foster City, CA).

### Bioinformatic and in silico DNA sequence analyses

Genomic sequences determined based on oligonucleotide coordinates from aCGH experiments were downloaded from the UCSC genome browser (NCBI build 37/hg19, http://genome.ucsc.edu) and assembled using Sequencher 4.8 software (Gene Codes Corporation, Ann Arbor, MI, USA). Interspersed repeat sequences were identified using RepeatMasker (http://www.repeatmasker.org).

To determine the occurrence of polymorphism in the minisatellite region in the general population, the Database of Genomic Variants: Structural Variation track in the UCSC genome browser was used. Orthologous VNTR sequences to the minisatellite sequence in other genomes were obtained using the convert and blat functions in the UCSC genome browser.

To compare and visualize syntenic sequences among different organisms, main repeated motifs were extracted and subjected to pairwise alignment. Repeats composing each sequence were aligned to form one set consisting of all existing motifs among the considered species. The resulting set was clustered into several groups using the gready clusterization algorithm: the most numerous motifs were assumed to be centers, while number of differences was assumed to be metric. One cluster contains motifs that differ from the center-motif with at most four point mutations and four point deletions.

Locations and sizes of VNTRs in the human genome, identified by Tandem Repeats Finder (TRF: http://tandem.bu.edu/trf/trf.submit.options.html) were downloaded from the Simple Tandem Repeats track in the UCSC genome browser [26]. Distances of VNTRs from the nearest centromere and telomere were calculated using the R statistical program (http://cran.r-project.org).

## Results

### Array CGH, FISH, Long-range PCR and DNA sequence analyses

**Figure 1:**
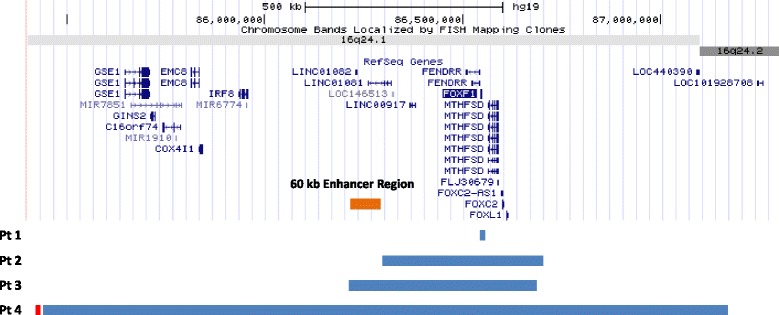
**Schematic representation of the 16q24.1 duplications in patients 1-4.** Genomic coordinates correspond to hg19 build of the human genome. Blue bars represent duplications. The distant 60 kb *cis*-regulatory region mapping 272 kb upstream of *FOXF1* is shown in orange and the minisatellite adjacent to the duplication in patient 4 is shown in red.

The 16q24.1 duplications involving *FOXF1* in patients 1-4 are shown in Figure 1.

#### Family 1

**Figure 2:**
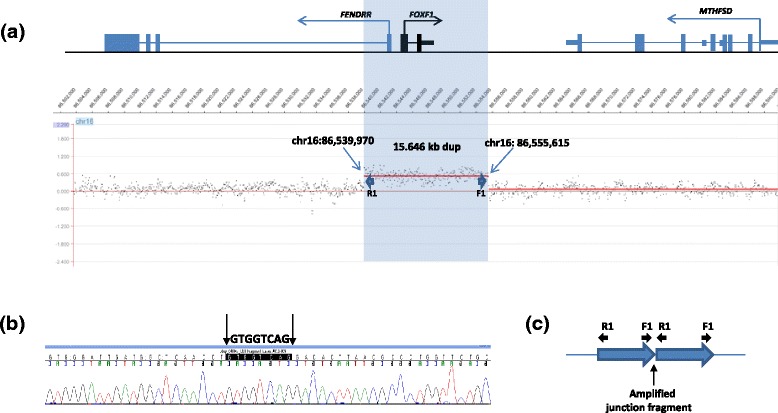
**Results of aCGH and DNA sequence analyses in patient 1. (a)** aCGH plot with the custom-designed NimbleGen 720K microarray showing duplication on chromosome 16q24.1. **(b)** Chromatogram of the DNA sequence of the junction fragment showing the 8 bp microhomology GTGGTCAG. **(c)** Schematic representation of the strategy used to amplify the duplication breakpoint junction fragment. The wildtype band is not amplified using this approach with the outward facing primers.

In patient 1, CMA with V8.1.1 OLIGO revealed a 4-109 kb duplication on chromosome 16q24.1 involving *FOXF1* and an ~1.09 Mb mosaic deletion of the *NF1* region on chromosome 17q11.2. The 16q24.1 duplication was also found by CMA in the patient’s father. *NF1* deletions are classified into type I caused by nonallelic homologous recombination (NAHR) during meiosis and type II arising from NAHR during mitosis and associated with a high frequency of somatic mosaicism [27,28]. Unfortunately, the resolution of our clinical array did not allow for distinguishing between these two kinds of deletions. Custom-designed high-resolution region-specific aCGH analyses demonstrated that the 16q24.1 duplication is ~15 kb in size (Figure 2a). FISH analysis in patient 1 with the BAC clone RP11-142O6 showed that the *NF1* deletion in 17q11.2 is mosaic and present in 37% of blood cells examined. Using long-range PCR (LR-PCR) with primers F1 and R1 (Additional file 1: Table S1), the proximal breakpoint of the 16q24.1 duplication was mapped at chr16:86,539,970-86,539,977 (hg19) and the distal breakpoint was mapped at chr16:86,555,608-86,555,615, defining a 15,645 bp tandem head-to-tail duplication. The duplication harbors the entire *FOXF1* gene, its promoter, and exon 1 of the lncRNA *FENDRR*. DNA sequence analysis of the junction fragment revealed 8 bp GTGGTCAG microhomology (Figure 2b). The distal breakpoint is located within a SINE/MIR repetitive element and the proximal breakpoint within a unique sequence. Schematic representation of the strategy used to amplify the duplication breakpoint junction fragment is shown in Figure 2c.

#### Family 2

**Figure 3:**
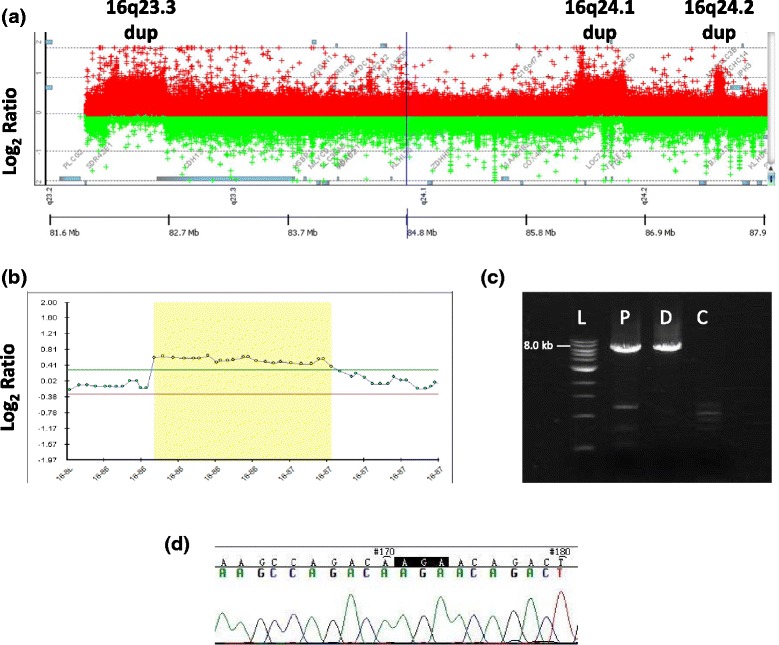
**Results of aCGH and DNA sequence analyses in patients 2 and 3. (a)** aCGH plot obtained using 4x180K microarray (Agilent) in patient 2 shows three duplicated regions in 16q23.3, 16q24.1, and 16q24.2. **(b)** aCGH plot from Illumina SNP-Array (Infinium® CytoSNP-850K showing duplication on chromosome 16q24.1 in patient 3’s daughter. **(c)** Duplication junction fragment visualized on 1% agarose gel in the proband (P) and her daughter (D), but absent in the control DNA (C). **(d)** Chromatogram of the DNA sequence of the junction fragment showing the 3 bp AGA microhomology.

In patient 2, CMA identified a copy-number gain of chromosome band 16q23.3 spanning 505-592 kb followed by another copy-number gain of the nearby 16q24.1 region spanning 265-327 kb and harboring *FOXF1*. Both duplications were inherited from the father. Custom-designed high-resolution aCGH analyses refined the duplication coordinates to chr16:82,215,062-82,728,565 (0.51 Mb; 16q23.3), involving an upstream portion of *CDH13* and chr16:86,286,094-86,714,315 (0.43 Mb; 16q24.1), harboring entire FOX gene cluster with *FOXF1, FOXC2*, and *FOXL1*. Additionally, custom-designed high-resolution aCGH analyses identified a third ~52 kb duplication at 16q24.2 (chr16:87,510,166-87,562,249) (Figure 3a).

#### Family 3

In patient 3, CMA identified a 417 kb copy-number gain of chromosome band 16q24.1 (chr16:86,211,060-86,628,524), encompassing 16 genes, including *FOXF1* (Additional file 1: Figure S2). G-banded chromosomal analysis was normal. Array CGH analysis in patient 3’s daughter revealed the same 16q24.1 duplication (chr16: 86,198,252-86,640,349); differences in coordinates are due to a different array platform (Figure 3b). Using LR-PCR with primers F3 and R3 (Additional file 1: Table S1), the proximal breakpoint of the 16q24.1 duplication was mapped at chr16:86,194,788-86,194,790 and the distal breakpoint at chr16:86,642,276-86,642,278, defining a 447,488 bp tandem head-to-tail duplication (Figure 3c). DNA sequence analysis of the junction fragment revealed 3 bp AGA microhomology (Figure 3d).

#### Family 4

**Figure 4:**
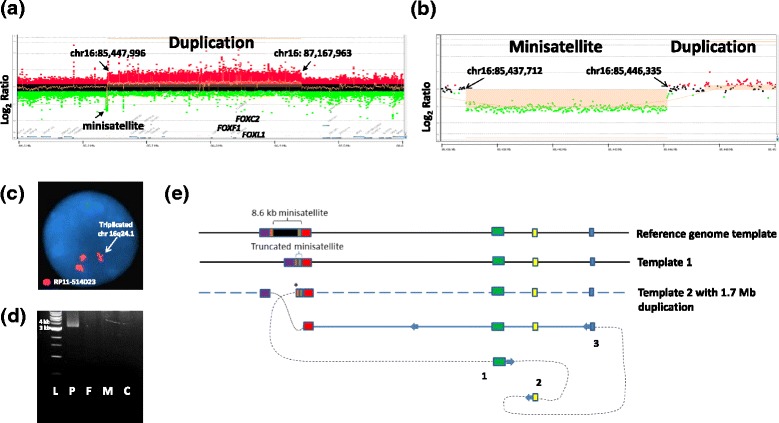
**Results of aCGH and DNA sequence analyses in patient 4. (a, b)** aCGH plot (4x180k Agilent microarray) showing the duplicated region in patient 3 in chromosome 16q24.1. **(c)** Result of FISH analysis showing the 16q24.1 duplication in the proband. **(d)** Duplication junction fragment visualized on 1% agarose gel in the proband (P), but absent in father (F), mother (M), and control DNA (C). **(e)** A proposed model of duplication formation mediated by the adjacent unstable minisatellite: 1, 2, and 3 represent template switches with 8 bp insertion (1) and microhomologies (2 and 3). Asterix (*) represents the initial DNA replication slippage event that might have triggered the formation of the described complex genomic rearrangement. Red boxes represent the MLT1D ERVL-MaLR repeat sequences flanking the minisatellite. Green and yellow boxes represent triplicated sequences.

In patient 4, 8 × 60K microarray revealed a 1.65-1.80 Mb 16q24.1 duplication. Our customized array enabled narrowing the duplication breakpoints to chr16:85,447,996-87,167,963, adjacent and distal to a large minisatellite (chr16:85,437,712-85,446,335; 8.6 kb) (Figure 4a, b). FISH analysis with the BAC clone RP11-514D23 confirmed the 16q24.1 duplication and excluded an insertional translocation (Figure 4c).

The duplication breakpoints were mapped using LR-PCR with primers F4 and R4 (Figure 4d, Additional file 1: Table S1). Sequence analysis of the breakpoint junction fragment revealed additional complexity, including truncated segments of the minisatellite sequence (chr16:85,437,697-85,446,384), an 8 bp insertion, and two other junctions with microhomologies (Additional file 1: Figure S3). Due to the highly repetitive nature of the minisatellite, it was not possible to sequence the breakpoints located within it.

### Analysis of non-human primates

LR-PCR applied across the tandem repeats in Chimp (*Pan troglodytes*) and Rhesus (*Macaca mulatta*) genomes revealed the evolutionarily polymorphic nature of the repeat (Additional file 1: Figure S4).

### Microsatellite analysis

Microsatellite analysis in patient 4 was informative for markers *D16S486, D16S520,* and *D16S3074* and showed that the duplication arose on the maternal chromosome (Table 1, Additional file 1: Figure S5).

**Table 1:** **Fragment analysis for microsatellite markers on 16q24.1 in patient 4 and his unaffected parents**

**Microsatellite marker**	**Genomic coordinates (hg19)**	**Patient 4’s genotype**	**Father’s genotype**	**Mother’s genotype**
*D16S539*	chr16:86,386,034-86,386,428	146/146	146/158	146/162
*D16S488*	chr16:86,386,286-86,386,641	236/236	236/248	236/252
*D16S486*	chr16:86,490,068-86,490,708	380/**384**	377/380	380/384
*D16S520*	chr16:86,516,112-86,516,335	195/**183**	181/195	183/183
*D16S3074*	chr16:87,084,745-87,085,073	188/**217**	188/211	188/217

Informative alleles inherited from the mother are shown in bold.

### Minisatellite-Bioinformatic and aCGH analyses

**Figure 5:**
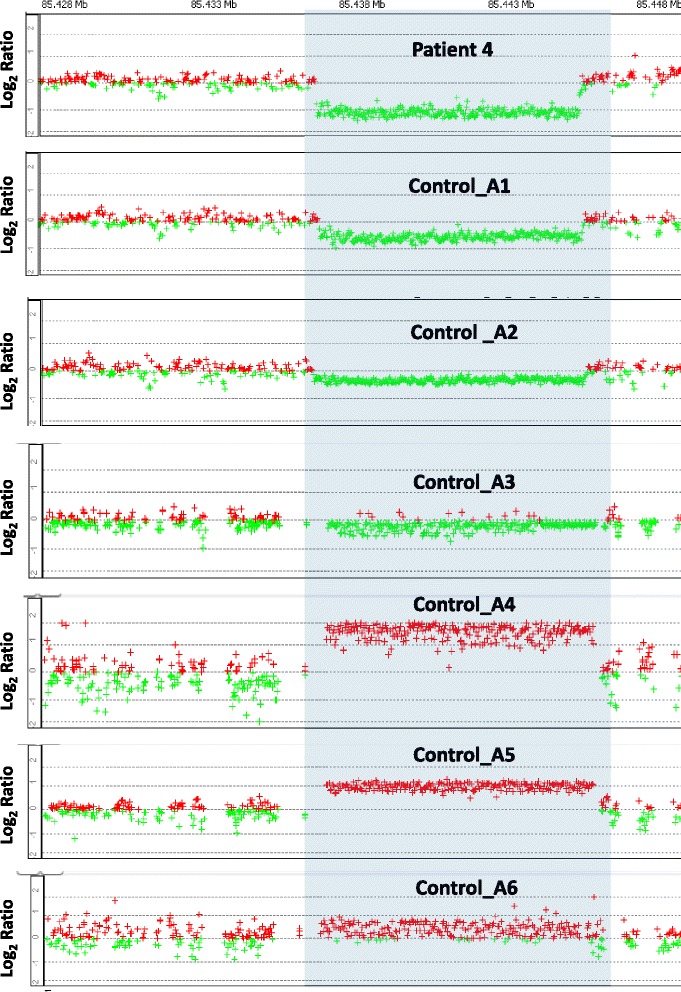
**High-resolution custom-designed region-specific Agilent CGH microarray analyses of the studied minisatellite in 16q24.1.** aCGH plot for patient 4 and 6 non-duplicated control samples run on 4x180k 16q24.1-specific Agilent microarray. Due to the repetitive nature of the minisatellite, contraction or expansion of the minisatellite shows decrease or increase in log ratios for all oligo probes in this region.

**Figure 6:**
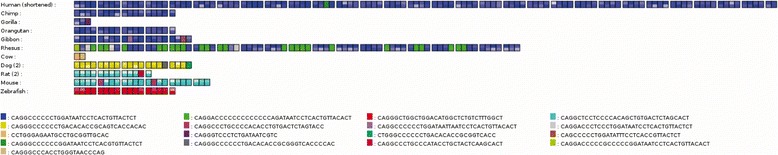
**Cross-species visualization of syntenic sequences of the 8.6 kb minisatellite in 16q24.1.** The figure presents the variation of motifs among different species and their conservation. Repeat sequences are represented as a row of multicolor strips and each strip maps to a particular group of motifs. The descending intensity of the color used to code the particular motif indicates the increasing number of differences from the motif corresponding to that color (mutational and deleterious differences are represented by upper and lower part of the strip, respectively). To increase the clarity of the figure, the human sequence was shortened to ~4.1 kb, and the rat and dog sequences are represented by one of the insertional translocation sequences (Table 2).

In the reference human genome, the large minisatellite is 8,688 kb in size, consisting of imperfect repeats of 33 bp sequence CAGGGCCCCCCGGATAATCCTCACTGTTACACT and is flanked by MLT1D ERVL-MaLR repeat sequences. Comparison of the aCGH results for this region in patient 4 with control samples run on high-resolution 16q24.1 arrays revealed the polymorphic nature of this tandem repeat (Figure 5, Additional file 1: Figure S6). In addition, several variants of this region are reported in the Database of Genomic Variants (DGV) (Additional file 1: Figure S7). Use of convert and blat functions in the UCSC genome browser showed the presence of orthologous VNTRs in several genomes across species (Table 2, Figure 6). Of note, in a few species, more than one homologous non-syntenic loci were identified, further suggesting their evolutionary instability.

**Table 2:** **Location of orthologous sequences to the 8.6 kb minisatellite across species**

**Species**	**Orthologous genomic coordinates**	**Other genomic locations**	**Genome build**
Human	chr16:85,437,697-85,446,384 (8688 bp)	N/A	hg19
Chimp	chr16(+):85,057,404-85,058,000 (597 bp)	chr6(−):171,902,631-171,903,434 (804 bp)	CSAC 2.1.4/panTro4
		chr16(−):87,565,917-87,566,290 (374 bp)	
Gorilla	chr16(+):75,975,460-75,975,529 (70 bp)	chr7(+):156,341,820-156,342,473 (654 bp)	gorGor3.1/gorGor3
Orangutan	chr16(+):73,141,772-73,142,390 (619 bp)	chr1(−):12,527,885-12,528,916 (1032 bp)	WUGSC 2.0.2/ponAbe2
Gibbon	chr2(+):158,448,609-158,449,280 (672 bp)	chr17(−):95,524,509-95,526,825 (2317 bp)	GGSC Nleu3.0/nomLeu3
		chr20(−):83,814,471-83,814,741 (271 bp)	
Rhesus	chr20(+):83,708,139-83,710,782 (2644 bp)	N/A	BGI CR_1.0/rheMac3
Cow	chr18(+):10,576,131-10.576,179 (49 bp)	N/A	Baylor Btau_4.6.1/bosTau7
Dog	chr5(+):67,222,084-67,222,093 (10 bp)	chr28(−):35,919,958-35,920,624 (667 bp)	Broad CanFam3.1/canFam3
Rat	chr14(+):3,644,385-3,645,161 (777 bp)	chr8(−):60,446,61-60,465,11 (1851 bp)	RGSC 5.0/rn5
		chr12(+):82,176,55-82,180,67 (413 bp)	
Mouse	chr2(+):167,074,771-167,075,545 (775 bp)	chr3(+):79,000,591-79,000,853 (263 bp)	GRCm38/mm10
		chr3(−):144,489,926-144,491,349 (1424 bp)	
		chr10(−):121,578,768-121,580,330 (1563 bp)	
Zebra fish	chr16(+):8,911,924-8,912,616 (693 bp)	chr4(+):44,314,003-44,315,463 (1461 bp)	Zv9/danRer7
		chr8(+):41,186,318-41,186,865 (548 bp)	
		chr15(−):11,992,633-11,992,908 (276 bp)

Genome-wide bioinformatics analyses showed that in contrast to VNTRs shorter than 1 kb, minisatellites longer than 1 kb cluster at pericentromeric and subtelomeric regions (Additional file 1: Figure S8). The distribution of VNTRs greater than 1 kb, 3 kb, and 5 kb in size across all chromosomes is shown in Additional file 1: Figure S9.

## Discussion

In humans, *FOXF1* expression is restricted to the fetal and adult lungs, prostate, and placenta. Unfortunately, we do not have RNA samples from these organs to inform on the expression levels of *FOXF1* in our patients. Hence, to determine whether *FOXF1* could be functionally over-expressed in the patients, we studied the involvement of its upstream regulatory elements in the duplicated segments, as well as their parental origin, given the incomplete paternal genomic imprinting of *FOXF1* in the human lungs [6,7].

Genomic duplications excluding regulatory elements of dosage-sensitive genes have been proposed to lead to inefficient transcription of the extra gene copy and lack of functional consequences. Amor et al. [29] reported an ~88 kb duplication at 14q12, encompassing the dosage-sensitive *FOXG1* gene in a father-son pair with isolated hemifacial microsomia. Neither the son nor the father exhibited ID or epilepsy. The authors questioned the pathogenicity of the increased dosage of *FOXG1*. However, this duplication did not include the distant *cis*-regulatory elements of *FOXG1* [30-33]. Moreover, a 7.4-kb *cis*-regulatory deletion disrupting conserved noncoding sequences and their interaction with the promoter of another FOX gene, *FOXL2*, mapping more than 280 kb apart, has been described as pathogenic for blepharophimosis, ptosis, and epicanthus inversus (BPES; OMIM 110100) [34]. This suggests that the presence of long-range regulatory elements could be a more general phenomenon common to the forkhead family FOX genes and highlights the importance of determining the exact location of the duplication breakpoints.

In family 1, the distant regulatory element upstream of *FOXF1* is not duplicated. The duplicated exon 1 and part of the 1^st^ intron of the *FENDRR* transcript is likely non-functional (Figure 2a), leaving the duplicated copy of *FOXF1* devoid of its distant upstream *cis*-regulatory element which maps 272 kb upstream (chr16:86,212,040–86,271,915) [6,7]. Additionally, given the fact that the duplication is inherited from the father, the increase of *FOXF1* dosage in patient 1 is probably minimal, if any. Similarly, the *FOXF1* duplication in patient 2 does not encompass the distant upstream *cis*-enhancer, and is paternally inherited. Hence, the increase in the expression level of *FOXF1* in patient 2 may also be minor. Conversely, the lung-specific enhancer region is duplicated in patients 3 and 4. However given the similarity in symptoms between patient 3 and her father, the duplication in patient 3 is likely paternally inherited and, the increase of *FOXF1* expression in patient 3’s lungs is likely minimal. In contrast, as the duplication in patient 3’s daughter and patient 4 arose on the maternal chromosome, the duplicated *FOXF1* copy in these patients is likely functional and associated with an increase of *FOXF1* expression in the lungs.

Along with mild dysmorphic features, patient 1 has one café-au-lait macule on his left flank and speech delay, typical features of Neurofibromatosis type 1 (NF1; OMIM 162200). Hence, speech delay and other abnormalities in the proband likely result from the *NF1* deletion [35] rather than from the *FOXF1* duplication. This notion is further supported by the fact that the father with isolated *FOXF1* duplication was reportedly healthy.

In addition to the *FOXF1* duplication on 16q24.1, patient 2 has two duplications on 16q23.3 and 16q24.2. The 16q23.3 duplication is also inherited from the father with bipolar disorder, suggesting that genes other than *FOXF1* may be responsible for the patient’s autistic and emotional disorders.

Recently, a patient harboring a complex *de novo* duplication-triplication rearrangement in 16q24.1-q24.3 was reported [36]. The patient presented with severe psychomotor disability, numerous dysmorphic features, and congenital malformations, including gut malrotation and gall bladder agenesis. The phenotype in the patient was attributed to the increased dosage of *FOXF1, FOXC2, ANKRD11, SPG7,* and *FANCA* in the duplicated/triplicated regions. Segregation of gastrointestinal abnormalities with *FOXF1* duplication in family 3 indicates that *FOXF1* is incompletely paternally imprinted only in the human lungs [6] and that the gastrointestinal symptoms in this family may be due to overexpression of *FOXF1* in the intestine.

Interestingly, maternal uniparental disomy of chromosome 16 usually associated with mosaic trisomy 16, and thus resulting in even higher dosage of *FOXF1* is associated with pulmonary hypoplasia, congenital heart defects, tracheosophageal fistula, gut malrotation, and renal agenesis in addition to intrauterine growth restriction [37], suggesting *FOXF1* expression higher than that in constitutional duplications may influence development of organs typically affected in patients with ACDMPV.

The presence of ID and speech delay in patient 4 may result from the duplication of other genes in the duplicated segment on 16q24.1. This notion is further supported by another Decipher patient 265650 with a *de novo* ~0.26 Mb duplication at chr16:85,678,461-85,942,847 (leaving *FOXF1* intact) and presenting with ID and delayed speech and language development. This duplication involves the *C16orf74, COX4I1, EMC8, GINS2, GSE1,* and *IRF8* genes that are also duplicated in patient 4. The absence of any lung defects in all four patients suggests that constitutional duplication of *FOXF1* is likely not pathogenic in the human lungs.

Interestingly, the duplication in patient 4 mapping distal and adjacent to a large minisatellite contains in its junction fragment, truncated segments of this minisatellite. We found this minisatellite is highly polymorphic in the general population, with many deletions and duplications reported in DGV as well as in several control DNA samples analyzed on our customized high-resolution 16q24.1 CGH microarrays (Figure 5), indicating that it contracts and expands. The orthologous VNTRs of this minisatellite in several species are much shorter and range from 597 bp in Chimp to 2,644 bp in Rhesus (Figure 6, Table 2), demonstrating its high instability during the evolution of the human genome. Amplification of this region in chimp and Rhesus genomes showed that, similar to human genome, this VNTR is polymorphic (Additional file 1: Figure S4).

Genome-wide bioinformatic analyses showed that when classified based on repeat length, the density of minisatellites greater than 1 kb in length is highest in the pericentromeric and subtelomeric regions. Conversely, VNTRs shorter than 1 kb in length are found to cluster primarily away from the pericentromeric regions (Additional file 1: Figure S8). PCR amplification of the minisatellite located at 16q24.1 in the mother of patient 4 revealed that it is truncated (~2 kb) when compared to the reference human genome (~8.6 kb). We suggest that the smaller size of this minisatellite in the mother of patient 4 might have undergone an incomplete heterochromatinization, predisposing it to a higher rate of DNA replication errors [38] and subsequent formation of the complex rearrangement via break-induced replication (BIR) or microhomology-mediated break-induced replication (MMBIR) [39,40]. We propose the microduplication arose pre-meiotically, as a mitotic DNA replication error in the patient 4’s mother (Figure 4e). A DNA replication slippage within the minisatellite led to the 8 bp GAGCAGCC insertion and subsequent strand invasion at chr16(-):86,978,360. A 2 bp GC microhomology mediated another template switch in the reverse direction from chr16(-):86,979,735 to chr16(+):87,102,896 and a 2 bp CA microhomology might have mediated another template switch in the forward direction from chr16(+):87,102,675 to chr16(+):87,168,469. This resulted in a triplication of the segments chr16(+):87,102,675-87,102,896 (221 bp) (yellow block) and chr16(-):86,978,360-86,979,735 (1375 bp) (green block), in addition to the duplication observed (Figure 4e). A high frequency of small deletions and insertions, likely originating from polymerase slippage events at breakpoint junctions of complex copy-number variants, have recently been reported in patients with *MECP2* duplications [41]. In addition, analyses of structural variation from the first sequenced personal genome [42,43] showed that minisatellites were responsible for 25.8% of medium-sized (<10 kb) structural variants [44]. Interestingly, intersection of VNTRs larger than 1 kb with uncertain CNV breakpoint regions (between minimum and maximum coordinates) smaller than 20 kb in size identified in 16,886 patients from the CMA database of 39,729 patients analyzed at MGL, revealed 156 unique CNVs, suggesting some of them might have also been mediated by long minisatellites (Additional files 1 and 2: Table S2).

## Conclusions

In aggregate, we describe 16q24.1 duplications involving *FOXF1* in four unrelated families with speech delay, ID, and gastrointestinal abnormalities. The lack of any pulmonary symptoms in these patients suggests relatively benign pediatric pulmonary consequences of *FOXF1* overexpression due to constitutional duplications. Discerning the effects of *FOXF1* overexpression or ectopic expression is of primary importance for any future work towards *FOXF1*-based gene therapies for ACDMPV and other disorders caused by *FOXF1* abnormal dosage. Given the findings in family 3 and the fact that *FOXF1* alterations were associated with intestinal abnormalities [2,4,36,45], we propose that patients with gut malrotation, pyloric or duodenal stenosis, or gall bladder agenesis should be tested for *FOXF1* mutations and CNVs.

Moreover, our analyses revealed an evolutionarily unstable and highly polymorphic minisatellite in 16q24.1. We propose that instability of minisatellites greater than 1 kb can lead to genomic structural variation due to DNA replication errors.

## Consent

Written informed consent approved by the Institutional Review Board (IRB) at BCM was obtained from patients 1, 2, 3 as well as patient 2’s father and patient 3’s daughter for publication of this report and any accompanying images. In case of minors, consent was obtained from their parent. Written informed consent for publication of this report and any accompanying images approved by the IRB at Saint Vincent de Paul Catholic Hospitals Association of Lille, Free Faculty of Medicine, Lille, France was obtained from patient 4 and both his parents.

## Additional files

Additional file 1:
**Supplementary Information.** Molecular and clinical analyses of 16q24.1 duplications involving *FOXF1* identify an evolutionarily unstable large minisatellite. Additional file 2: Table S2. Intersection of VNTRs (> 1 kb) with uncertain CNV breakpoint regions (< 20 kb) from the CMA database at MGL.
