# Supplementary material for: Molecular and clinical analyses of 16q24.1 duplications involving FOXF1 identify an evolutionarily unstable large minisatellite
Source: BMC Med Genet. 2014 Dec 4;15:128. doi: 10.1186/s12881-014-0128-z (PMC4411736; doi:10.1186/s12881-014-0128-z)
Supplement: Additional file 1: — Supplementary Information. Molecular and clinical analyses of 16q24.1 duplications involving FOXF1 identify an evolutionarily unstable large minisatellite. [file 12881_2014_128_MOESM1_ESM.docx]

**Supplementary data**

**Molecular and clinical analyses of 16q24.1 duplications involving *FOXF1* identify an evolutionarily unstable large minisatellite**

Avinash V. Dharmadhikari^1,2,*^, Tomasz Gambin^2,*^, Przemyslaw Szafranski^2,*^, Wenjian Cao^2^, Frank J. Probst^2^, Weihong Jin^2^, Ping Fang^2^, Krzysztof Gogolewski^3^, Anna Gambin^3,4^, Jaya K. George-Abraham^5^, Sailaja Golla^6^, Francoise Boidein^7^, Benedicte Duban-Bedu^8^, Bruno Delobel^8^, Joris Andrieux^9^, Kerstin Becker^10^, Elke Holinski-Feder^10^, Sau Wai Cheung^2^, Pawel Stankiewicz^1,2*^

^1^Interdepartmental Program in Translational Biology & Molecular Medicine, Baylor College of Medicine, Houston, TX; ^2^Department of Molecular and Human Genetics, Baylor College of Medicine, Houston, TX; ^3^Institute of Informatics, University of Warsaw, Warsaw, Poland; ^4^Mossakowski Medical Research Center, Polish Academy of Sciences, Warsaw, Poland; ^5^Specially for Children, Dell's Children's Medical Center, Austin, TX; ^6^Departments of Pediatrics and Neurology, University of Texas Southwestern Medical Center, Dallas, TX; ^7^Neuropediatrics Service, ^8^Cytogenetics Service, Saint Vincent de Paul Catholic Hospitals Association of Lille, Free Faculty of Medicine, Lille, France; ^9^Cytogenetics Service, University Hospital, Lille, France, ^10^Medical Genetics Center, Munich, Germany. * equal contribution

**Table S1: PCR primers used to amplify the junction fragments.**

| Patient 1 | F1 | 5’-GACCTCTGCGATTTATGGACATCAAAAAGA-3’ |
| --- | --- | --- |
|  | R1 | 5’-CCTACCAAGTCAGTATAATTTCCCTCCTCATT-3’ |
| Patient 3 | F3 | 5’-CCACTAGGTAGTCTCTGGCATATGTTCTATTC-3’ |
|  | R3 | 5'-GATTTGTCTCCATCAACCAGTTTAGCA-3' |
| Patient 4 | F4 | 5’-ACCTCAGCTAGTTGCCCTTCATCTATTCTTC-3’ |
|  | R4 | 5’-GTGTCTAGCTTGACTCCTCATCCCATAGAC-3’ |

**Table S2:** A list of 156 unique CNVs identified in 16,886 patients from the CMA database of 39,729 patients analyzed at MGL after intersection of VNTRs larger than 1 kb with uncertain CNV breakpoint regions (between minimum and maximum coordinates) smaller than 20 kb in size.

**Family 3**

**Family 2**

Gastrointestinal defects,

16q24.1 dup

Gastrointestinal defects,

16q24.1 dup

Mood and anxiety disorder,

16q24.1 dup,

16q23.3 dup

Bipolar disorder,

16q24.1 dup,

16q23.3 dup

Gastrointestinal defects

16q24.1 dup?

**Figure S1: Pedigree Analysis for families 2-and 3.** Pedigree of patients 2 and 3 and their respective family members showing inheritance of 16q24.1 duplications and associated phenotypes.


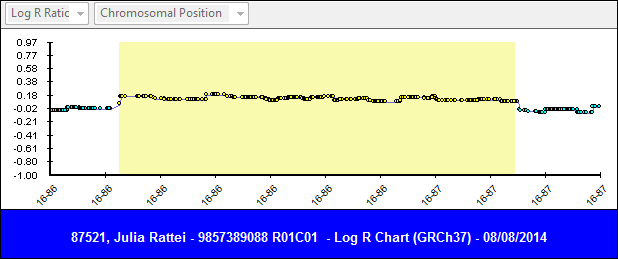


**Log_2_ Ratio**

**16q24.1 dup**

**Figure S2**: **Results of** **aCGH in patient 3.** aCGH plot from Oligonucleotide array (Cytochip v1.0 180K) showing duplication on chromosome 16q24.1 in patient 3

**
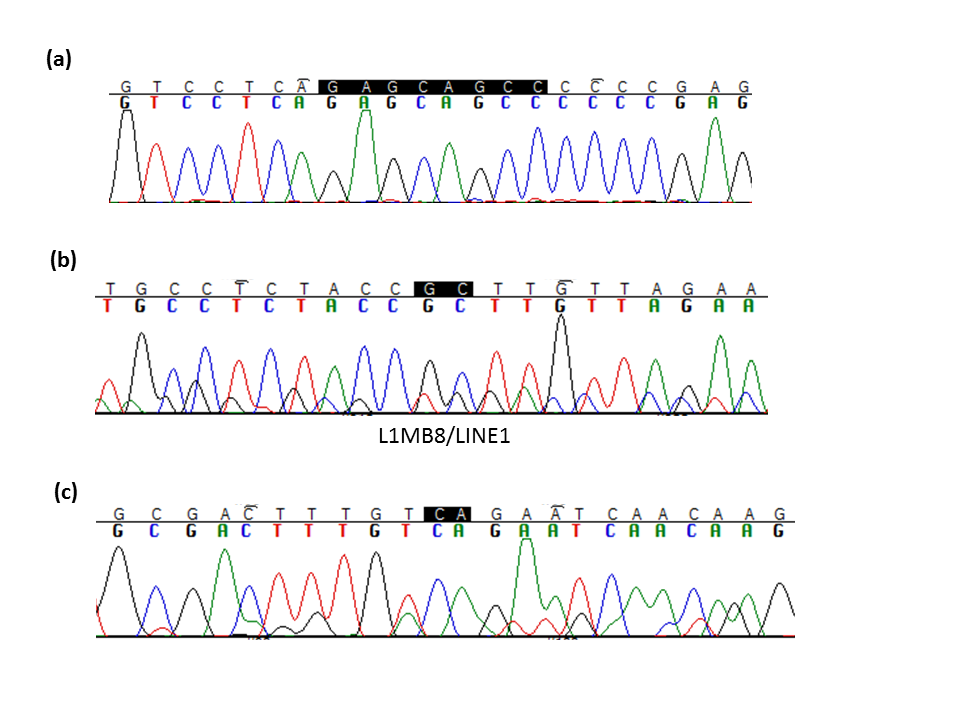
**

**Figure S3: Chromatograms of the DNA sequences of three junctions in the complex head-to-tail duplication in patient 4 showing short insertion and two microhomologies.** (a) 8 bp GAGCAGCC insertion in junction fragment (b) 2 bp GC microhomology mediating a template switch in the reverse direction from chr16(-):86,979,735 to chr16(+):87,102,896. The breakpoint is located in within a L1MB8/LINE1 repetitive element. (c) 2 bp CA microhomology mediating a template switch in the forward direction from chr16(+):87,102,675 to chr16(+):87,168,469.The breakpoint is located in a unique sequence.


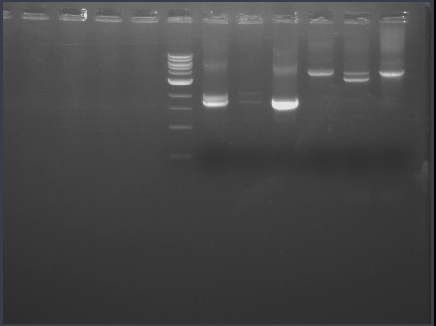


C1 C2 C3 R1 R2 R3

L

**Figure S4: Polymorphic nature of orthologous minisatellite regions in Chimp and Rhesus genomic DNA samples.** The orthologous VNTR sequences in the Chimp (samples C1,-C3) and Rhesus (samples R1-R3) genomes were amplified using LR-PCR; L-1kb ladder. Amplification of multiple bands suggests that this VNTR is polymorphic both in the Chimp and Rhesus genomes.

**D16S486**


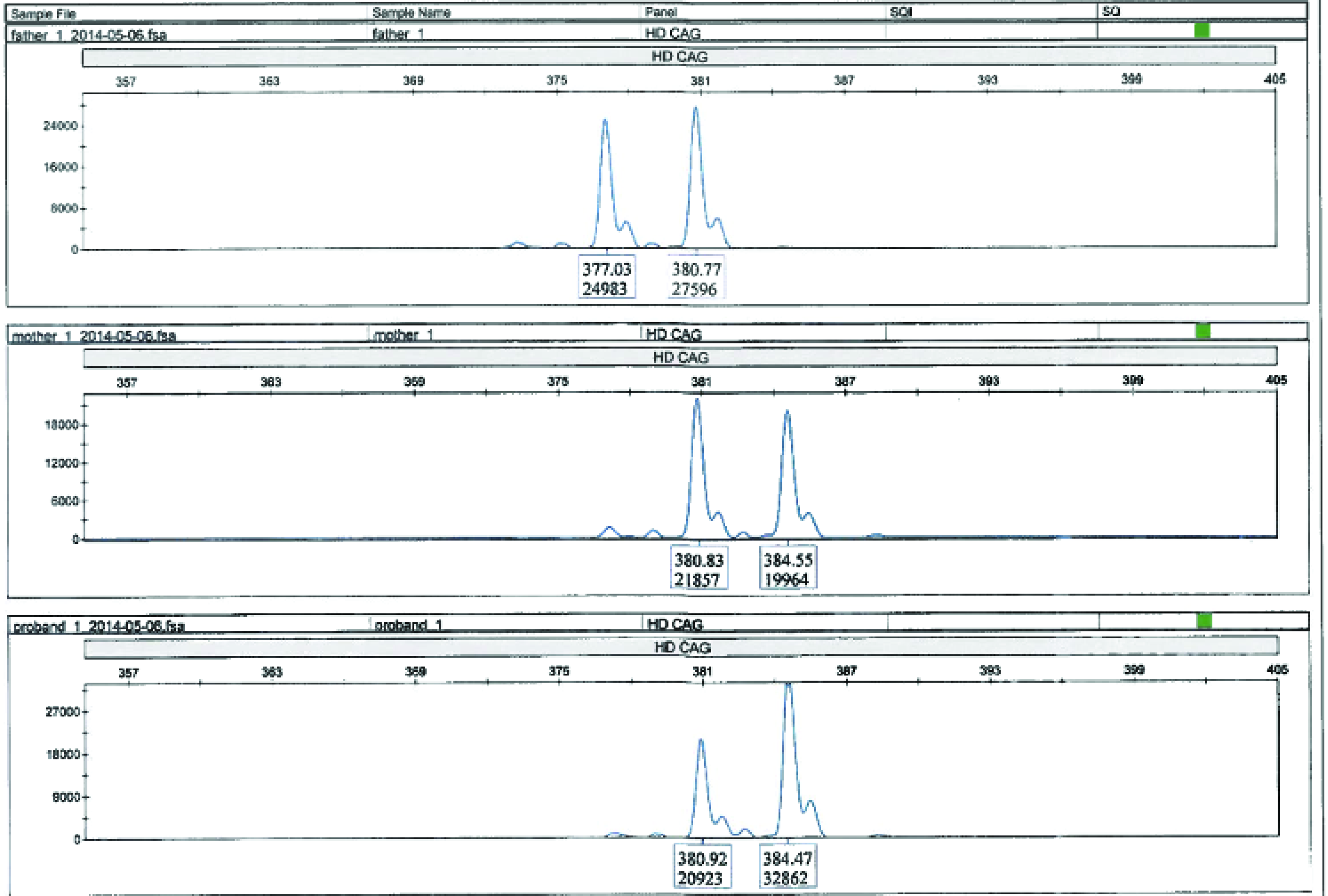


**Father**

**Mother**

**Patient 3**

**Figure S5: Microsatellite analysis showing maternal origin of duplication in patient 3.** Microsatellite analysis showing higher relative peak height of the allele (384) that was inherited from the mother compared to the height of the allele (380) inherited from the father.

**
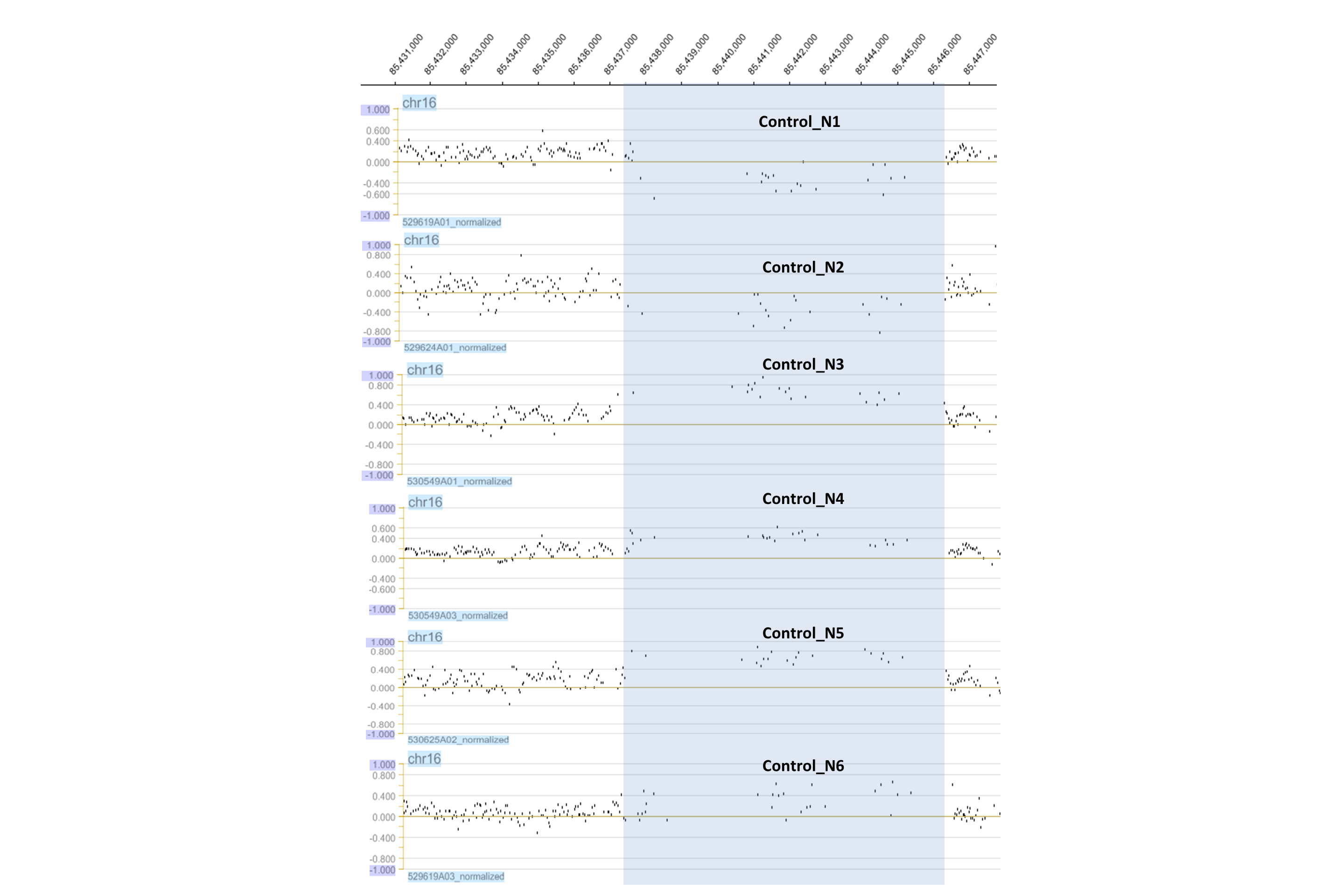
**

**Figure S6:** **High-resolution 16q24.1 NimbleGen aCGH analyses of the described minisatellite.** aCGH plot for six non-duplicated samples run on 3x720k 16q24.1 specific NimbleGen microarray. Due to the repetitive nature of the minisatellite, contraction or expansion of the minisatellite shows decrease or increase in log ratios for all oligo probes in this region.

**
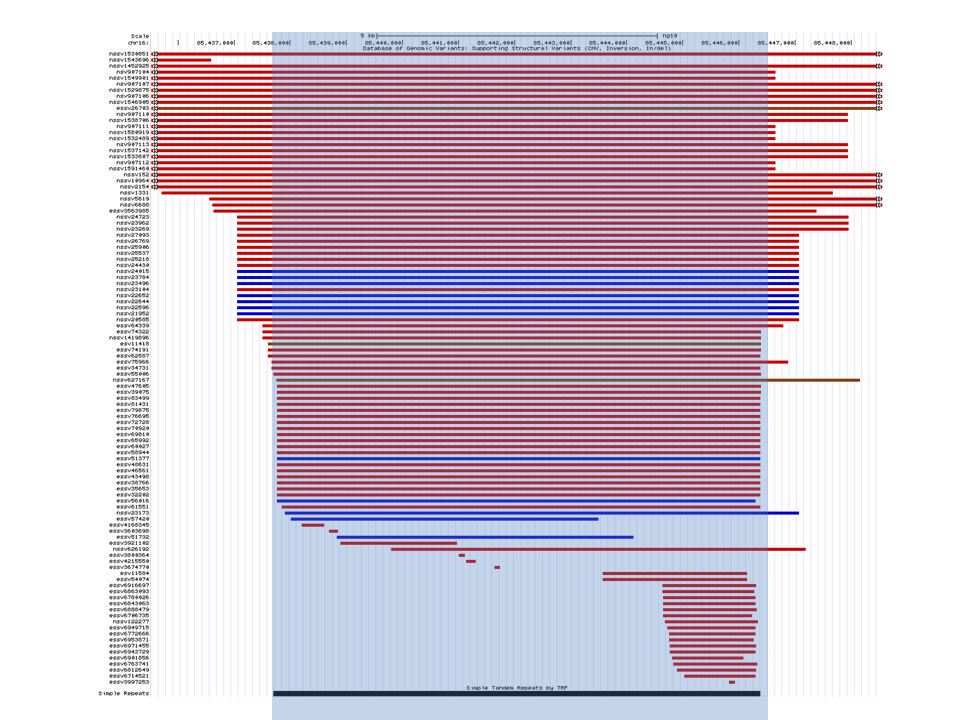
**

**Figure S7: Deletions and duplications in the 8.6 kb minisatellite reported in DGV.** Deletions are shown in red and duplications are shown in blue. Combination of custom designed 244K CGH microarray (Agilent), 42M CGH microarray (NimbleGen) and HiSeq2000 (Illumina) platforms were used to detect these copy-number variations in the general population. The black bar at the bottom represents the minisatellite.

**
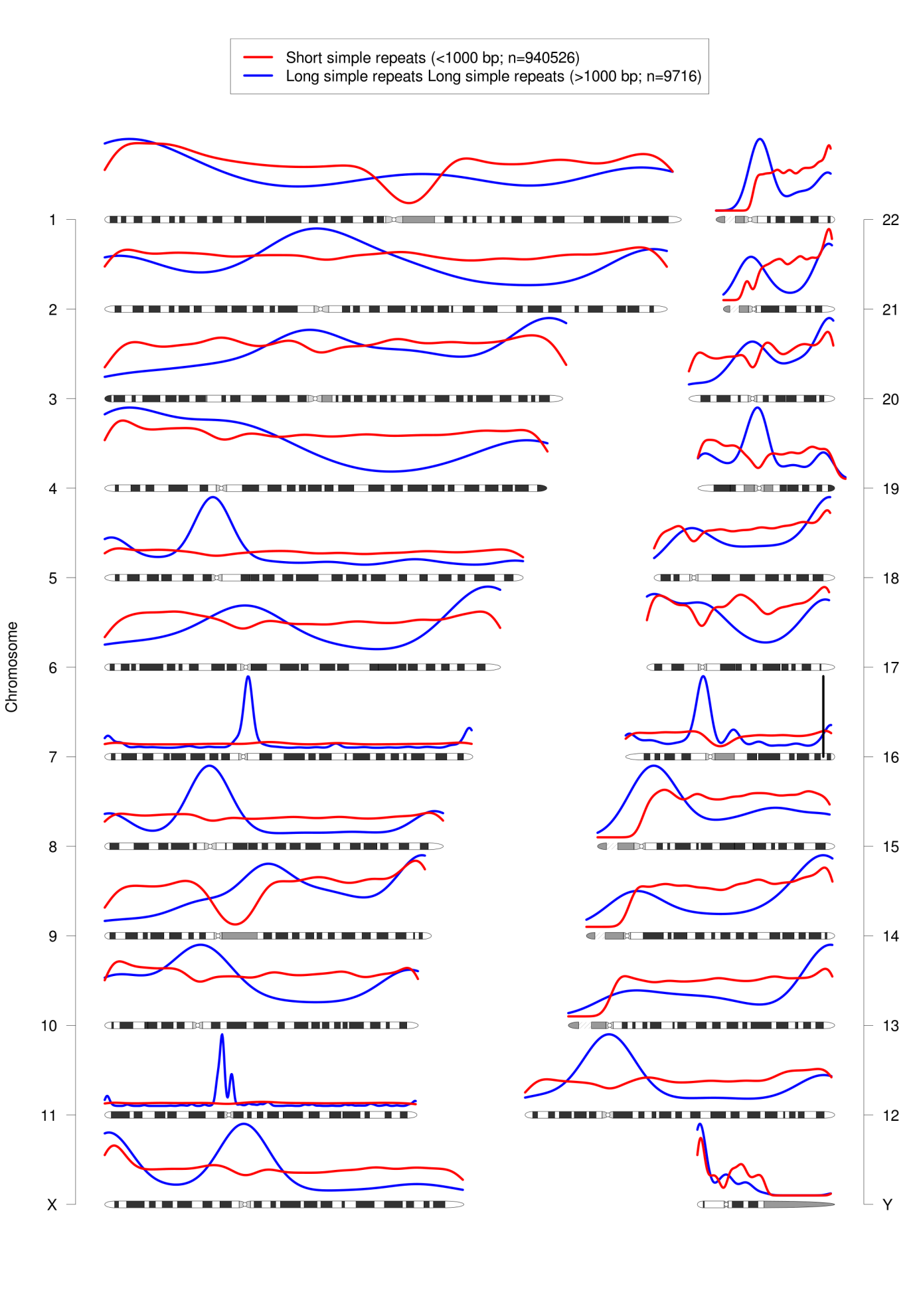
**

**Figure S8: Density of VNTRs across 22 autosomes and X and Y chromosomes.** Red lines represent VNTRs less than 1 kb in length and blue lines represent VNTRs greater than 1 kb in length. The vertical black bar shows the location of the minisatellite in 16q24.1.


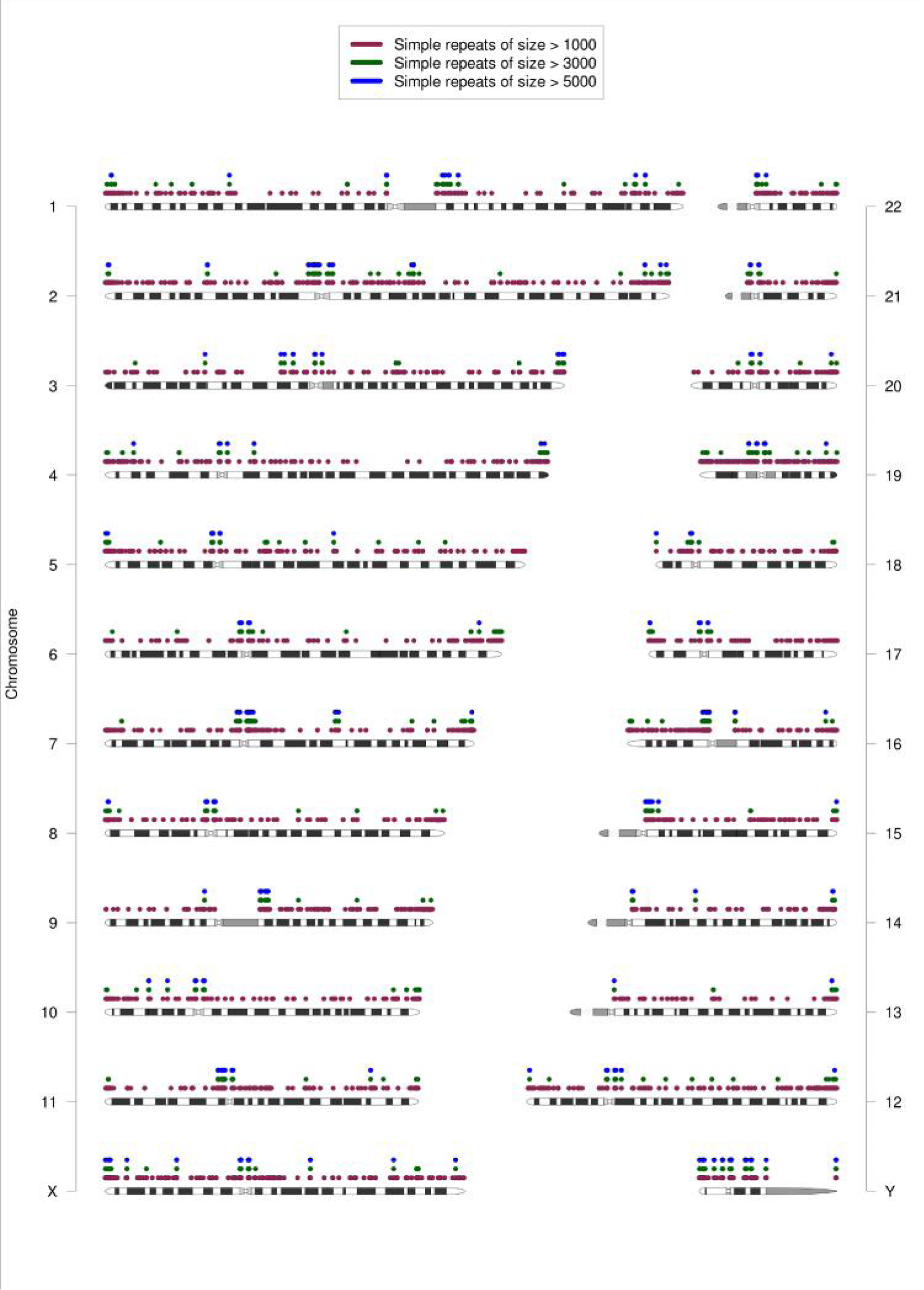


**Figure S9: Distribution of large simple VNTRs in the human genome.** Ideogram showing distribution of large simple VNTRs across 22 autosomes and X and Y chromosomes. Purple, green, and blue dots represent VNTRs greater than 1 kb, 3 kb, and 5 kb in length, respectively.
